# Asymptomatic Versus Symptomatic Alzheimer's Disease Neuropathology: A Systematic Review of Differences Reported in Post‐Mortem Studies

**DOI:** 10.1111/nan.70077

**Published:** 2026-05-08

**Authors:** Thiago Guilherme Rêgo Barros, Laura Barbosa Grossi, Maria Letícia da Silva Campos, Israel Bem‐Hur Netto Cardoso, Antônio Lúcio Teixeira, Aline Silva de Miranda, Eliana Cristina de Brito Toscano

**Affiliations:** ^1^ Laboratório Integrado de Pesquisas em Patologia, Departamento de Patologia, Faculdade de Medicina Universidade Federal de Juiz de Fora Juiz de Fora Brazil; ^2^ The Glenn Biggs Institute for Alzheimer's & Neurodegenerative Diseases, Lozano Long School of Medicine University of Texas Health Science Center at San Antonio San Antonio Texas USA; ^3^ Laboratório de Neurobiologia “Conceição Machado”, Departamento de Morfologia, Instituto de Ciência Biológicas Universidade Federal de Minas Gerais Belo Horizonte Brazil

**Keywords:** Alzheimer's disease, asymptomatic Alzheimer's disease, cognition, dementia, neuropathology, pathophysiology, post‐mortem examination, systematic review

## Abstract

A subset of individuals exhibit substantial Alzheimer's disease (ad) neuropathology but remain asymptomatic (ASYMAD). The biological processes underlying this phenomenon remain poorly understood. We searched the PubMed/MEDLINE, Web of Science and CAPES databases to synthesise post‐mortem brain tissue findings that differ between symptomatic and asymptomatic ad. Thirty‐four studies met the predefined eligibility criteria. Multiple reports described lower levels of oligomeric Aβ and p‐tau in ASYMAD, with reduced accumulation at synapses. Consistent with synaptic resilience, ASYMAD showed preserved expression of markers and proteomic signatures linked to synaptic function, homeostasis and plasticity. Further analyses supported preserved parenchymal architecture and dendritic and axonal morphology in ASYMAD. Some authors also described compensatory adaptations, including neuronal hypertrophy, increased neurogenesis and cellular antioxidant responses in ASYMAD brains. Additional findings indicated partial resistance to disruption of brain glucose, polyunsaturated fatty acid and one‐carbon/polyamine metabolism. Glial and immune findings suggested a profile characterised by increased glial reactivity in specific regions and disease stages, alongside preserved glial homeostasis and lower cytokine expression in other contexts. In conclusion, although this review identified multiple processes potentially associated with the ASYMAD phenotype, the current evidence remains largely descriptive. Additionally, this synthesis is limited by substantial methodological heterogeneity and inconsistencies in data reporting. Future research should prioritise standardised clinicopathological definitions, pathology stage matching between ASYMAD and symptomatic AD groups, systematic assessment of mixed pathologies and further replication of exploratory findings to better elucidate the mechanisms underlying clinical resilience to ad neuropathology.

## Summary

- A subset of individuals with Alzheimer's disease neuropathology does not exhibit symptoms.
- We reviewed post‐mortem studies comparing brain tissue findings in symptomatic and asymptomatic Alzheimer's disease.
- Current evidence suggests differences in proteinopathy profiles, neuronal integrity, metabolic pathways and glial signatures.
- Heterogeneous clinicopathological definitions and incomplete data reporting limit cross‐study comparability and mechanistic inference.
- Further investigation is needed to validate candidate mechanisms underlying clinical resilience to Alzheimer's disease neuropathology.

## Graphical abstract

Graphical summary of post‐mortem brain tissue findings that differ between symptomatic and asymptomatic ad. Current evidence suggests reduced accumulation of Aβ and p‐tau oligomers, particularly at synapses, preserved neuronal and synaptic integrity and metabolic activity and a dynamic profile of glial and immune responses in ASYMad. Created with BioRender in https://BioRender.com/qr041v2. Abbreviations: ad, Alzheimer's disease; ASYMAD, asymptomatic AD; Aβ, amyloid‐beta; NFT, Neurofibrillary tangles; p‐tau, phosphorylated tau.

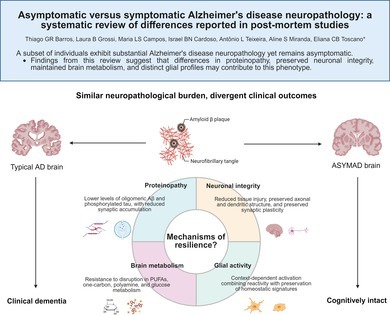

**Abbreviations**
ad
: Alzheimer's disease
ADNC: Alzheimer's disease neuropathologic change
APOE: apolipoprotein E
ASYMAD: asymptomatic individuals with Alzheimer's disease neuropathology
Aβ: amyloid‐beta
CERAD: Consortium to Establish a Registry for Alzheimer's Disease
LATE‐NC: limbic‐predominant age‐related TDP‐43 encephalopathy neuropathologic change
NFTs: neurofibrillary tangles
NIA‐AA: National Institute on Aging and the Alzheimer's Association
PRISMA: Preferred Reporting Items for Systematic Reviews and Meta‐Analyses
TDP‐43: TAR DNA‐binding protein 43

## 1 Introduction

Alzheimer's disease (ad) is a neurodegenerative disorder that leads to cognitive impairment, functional decline and neuropsychiatric symptoms [1, 2]. ad affects more than 35.6 million individuals and is the leading cause of dementia worldwide [3, 4]. In vivo diagnosis integrates clinical assessment, cognitive testing and neuroimaging [5]. A definitive diagnosis has traditionally required post‐mortem neuropathological examination [5, 6]. The neuropathological hallmarks of ad are extracellular neuritic plaques composed of 40–42 amyloid‐beta (Aβ) peptides and intracellular neurofibrillary tangles (NFTs) formed of phosphorylated tau (p‐tau) [7, 8].

Two widely used systems for evaluating these lesions are the Consortium to Establish a Registry for Alzheimer's Disease (CERAD) criteria and the Braak staging system [9, 10]. CERAD grades the density of neuritic plaques in the neocortex as none (C0), sparse (C1), moderate (C2) or frequent (C3) and allows an age‐adjusted interpretation of plaque burden (0/A/B/C) [9]. Braak staging describes the topographical progression of NFTs across three stages: I/II (entorhinal), III/IV (limbic) and V/VI (neocortical) [10]. Guidelines from the National Institute on Ageing and the Alzheimer's Association (NIA‐AA) integrate these measures with Thal phases to characterise ad neuropathologic change (adNC) and estimate the likelihood that dementia is attributable to ad [11].

In recent years, biomarkers reflecting amyloid and tau pathology have been increasingly incorporated into clinical practice to support the diagnosis of ad [12]. A 2024 NIA‐AA framework proposed that detection of abnormal core 1 biomarkers (A+T+) is sufficient to define biologic AD (e.g., amyloid positron emission tomography, cerebrospinal fluid measures of Aβ40/42 and total or p‐tau and blood levels of p‐tau217) [13, 14]. Growing evidence describes individuals who test positive for these biomarkers but do not exhibit symptoms [15, 16].

Post‐mortem evidence has also identified individuals with substantial ADNC at autopsy despite no antemortem evidence of dementia. In the National Alzheimer's Coordinating Center autopsy database, 252 of 4889 individuals with moderate or frequent CERAD scores (C2–C3) were clinically unimpaired, of whom 186 (74%) also had Braak stages III–VI [13, 17]. Corroborating evidence from a combined analysis of autopsies from the Adult Changes in Thought, Nun, Honolulu‐Asia Ageing and Oregon Brain Ageing studies reported that up to 60% of cognitively unimpaired participants exhibited varying degrees of adNC, often accompanied by mixed pathologies [18, 19].

Together, these findings support the existence of mechanisms that contribute to clinical resilience to AD neuropathology [15]. However, evidence on the cellular and molecular substrates underlying this phenomenon remains fragmented. Here, we systematically review studies comparing brain tissue findings in individuals with substantial adNC at autopsy who remained asymptomatic (ASYMad) with those in individuals with symptomatic AD. We aimed to identify biological processes associated with the ASYMAD phenotype, thereby generating hypotheses that may inform future mechanistic research and, ultimately, therapeutic strategies aimed at delaying or preventing progression to symptomatic AD.

## 2 Methods

The protocol for this review was registered in PROSPERO (CRD42024568239). Reporting followed the Preferred Reporting Items for Systematic Reviews and Meta‐Analyses (PRISMA) 2020 statement [20]. We used the PICO framework to formulate the research question and define the eligibility criteria [21].

### 2.1 Data Sources and Search Strategy

We conducted a systematic search to identify original studies investigating biological processes that may contribute to the absence of symptoms despite substantial ADNC at autopsy. Searches were conducted in PubMed/MEDLINE, Web of Science and CAPES from database inception to February 2026, using the following terms: ([Alzheimer's disease] OR [dementia]) AND ([cognitive resilience] OR [resistance to dementia]) AND ([asymptomatic Alzheimer's disease] OR [nondemented individuals]). Records were imported into Rayyan QCRI, and duplicates were removed [22]. Two independent reviewers (T.G.R.B. and L.B.G.) screened titles, abstracts and full texts against predefined eligibility criteria. Disagreements were resolved by consensus with a third reviewer (E.C.B.T.). We also conducted backward citation tracking to identify potentially eligible studies that may not have been retrieved during the initial search. Additional records identified through citation searching underwent the same selection procedures described above.

### 2.2 Eligibility Criteria

We included full‐text, peer‐reviewed original studies in which the primary analyses were performed on post‐mortem human brain tissue, aiming to identify cellular and molecular features associated with clinical resilience to sporadic ad‐related dementia. Studies were eligible if they reported comparisons between individuals described in the original reports as cognitively unimpaired yet meeting neuropathological criteria consistent with ad at autopsy, and individuals reported to have both a pathological and a clinical diagnosis of ad. Neuropathological characterisation was required, including assessment according to the CERAD and Braak criteria [9, 10]. Exclusion criteria included non‐English‐language publications, experimental models, reviews and case reports.

### 2.3 Data Extraction and Synthesis

Data were independently extracted by two reviewers (T.G.R.B. and L.B.G.) into a standardised spreadsheet. Variables included the demographic and clinicopathological characteristics of the groups, the brain regions examined, the analytical approaches used and the main findings. We also recorded the following potential confounders: post‐mortem interval, apolipoprotein E (APOE) genotype and copathologies. When group‐level data were not reported, but sufficient information was available, we derived summary measures, such as means and proportions, to standardise data presentation. We conducted a structured narrative synthesis, grouping findings into the biological categories of proteinopathy, neuronal integrity, brain metabolism and glial and immune signatures. Only findings relevant to our objective were extracted. We highlighted results that were consistent across independent studies and noted gaps that limited cross‐study comparability and certainty of the evidence.

### 2.4 Risk of Bias Assessment

Methodological quality was assessed using the Newcastle–Ottawa Scale [23, 24]. Two independent reviewers (T.G.R.B. and L.B.G.) assessed three domains: (i) selection, comprising four items evaluating case definition and representativeness, and the selection and definition of controls; (ii) comparability, comprising two items assessing matching or adjustment for key confounders, such as age and additional factors; and (iii) exposure, comprising three items assessing exposure ascertainment, consistency of ascertainment across groups and nonresponse rate. Each study was rated across the nine items and assigned a star score. As an a priori decision, studies scoring 0–4 stars were classified as being at high risk of bias, those scoring 5–7 as being at moderate risk and those scoring 8–9 as being at low risk (Table S1). Discrepancies were resolved by consensus with a third reviewer (E.C.B.T.).

## 3 Results

### 3.1 Study Selection

**FIGURE 1:**
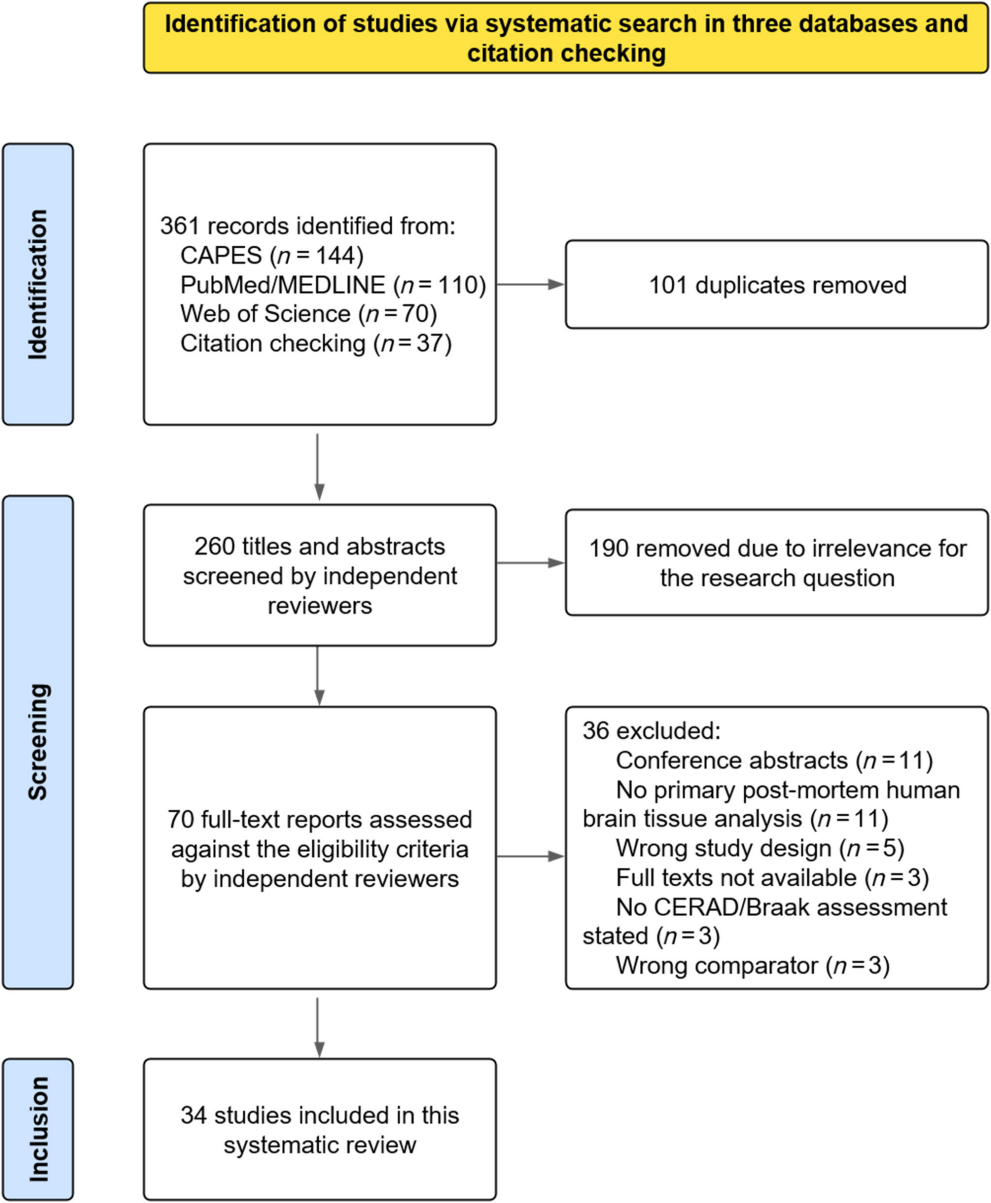
PRISMA flowchart of study selection. Flow diagram summarising the study selection process. ‘No primary analysis of post‐mortem human brain tissue’ included studies based on positron emission tomography or other imaging modalities, cerebrospinal fluid biomarkers and other nontissue approaches. Under ‘Wrong study design’, there were two case series, one review, one protocol and one preprint. ‘Wrong comparator’ included one study comparing ASYMAD with mild cognitive impairment, one comparing ASYMAD with healthy controls and one without a clearly defined ASYMAD group. All conference abstracts and posters were screened for subsequent publication as full‐text reports. Abbreviations: ASYMAD, asymptomatic Alzheimer's disease; PRISMA, Preferred Reporting Items for Systematic Reviews and Meta‐Analyses.

Figure 1 illustrates the PRISMA flow diagram for study selection. A total of 361 studies were initially identified. After deduplication, 260 records were screened for relevance to the research question. Seventy full‐text reports were assessed for eligibility, and 34 manuscripts met the criteria for inclusion in the final synthesis.

### 3.2 Study Characteristics

The included studies were published up to 2025 and represented 1337 post‐mortem brain samples. Almost all samples were derived from longitudinal cohorts in the United States, mainly, the Baltimore Longitudinal Study of Ageing, the Religious Orders Study and Memory and Ageing Project, the Oregon Brain Bank/Oregon Health & Science University and the NIH‐sponsored Layton Ageing and ad Center [25, 26, 27, 28, 29]. Key demographic and clinicopathological characteristics are summarised in Table 1.

**TABLE 1:** Main demographic and clinicopathological characteristics.

Ref.	Groups	Sample (F/M)	Age, years	Clinical instruments	ε4 carriers, *n* (%)	CERAD (range)	Braak (range)	Brain copathologies, *n*
[30]	CT	8 (2/6)	80.0 (2.0)	NR	0	0^a^	II^a^	NR
	ASYMAD	8 (2/6)	82.0 (3.0)	NR	3 (38%)	B^a^	IV^a^	
	AD	8 (2/6)	81.0 (2.0)	NR	6 (76%)	C^a^	VI^a^	
[31]	CT	14 (NR)	75.2 (9.8)	CDR = 0	NR	0	0–III	NR
	ASYMAD	4 (NR)	79.5 (5.9)	CDR = 0	NR	A	II–IV	NR
	Mild AD	9 (NR)	86.8 (6.1)	CDR = 0.5	NR	A–B	II–V	NR
	Severe AD	4 (NR)	73.8 (11.4)	CDR 3	NR	B–C	IV–V	NR
[32]	CT	11 (1/10)	81.4 (8.4)	MMSE ≥ 26	NR	0–A	I–IV	Cerebrovascular: 3
	ASYMAD	8 (1/7)	84.1 (8.4)	MMSE ≥ 26	NR	B–C	II–IV	Cerebrovascular: 1
	AD	14 (7/7)	82.9 (9.5)	MMSE < 25	NR	B–C	III–VI	None
[33]	CT	9 (0/9)	82.4 (10.8)	CDR = 0	NR	0	0–IV	Excluded cases with significant non‐AD brain lesions
	ASYMAD	9 (0/9)	85.0 (9.4)	CDR = 0	NR	B–C	II–IV	
	MCI	9 (0/9)	86.1 (9.9)	CDR = 0.5	NR	A–B	II–IV	
	AD	8 (0/8)	91.3 (5.8)	CDR ≥ 1	NR	B–C	II–VI	
[34]	CT	15 (2/13)	84.0 (2.2)	MMSE > 27	NR	0	0–IV	Excluded cases with significant non‐AD brain lesions
	ASYMAD	15 (3/12)	87.2 (2.1)	MMSE > 27	NR	B–C	0–IV	
	MCI	15 (3/12)	87.0 (2.2)	MMSE = 19–29	NR	A–B	0–IV	
	AD	15 (6/9)	92.0 (1.3)	MMSE = 4–26	NR	B–C	II–VI	
[35]	CT	13 (13/0)	83.3 (0.4)	MMSE = 28^b^	0%^c^	0	0–II	Excluded cases with significant non‐AD brain lesions
	ASYMAD	10 (10/0)	87.6 (0.9)	MMSE = 28^b^	5%^c^	A–C	III–V	
	MCI	5 (5/0)	87.8 (1.2)	MMSE = 26^b^	50%^c^	B–C	III–V	
	AD	10 (10/0)	87.7 (0.8)	MMSE = 3^b^	25%^c^	B–C	IV–VI	
[36]	CT	14 (4/10)	79.8 (9.1)	NR	NR	NR	NR	NR
	ASYMAD	10 (4/6)	86.6 (5.3)	NR	NR	C2–3	II–IV	NR
	AD	33 (18/15)	79.9 (6.9)	NR	NR	NR	NR	NR
[37]	ASYMad	8 (2/6)	92.8 (3.3)	MMSE > 24	2 (25%)	C2–3	III–V	Excluded cases with significant non‐AD brain lesions
	AD	6 (5/1)	94.2 (2.6)	MMSE 5–19	3 (50%)	C2–3	IV–VI	
[38]	CT	10 (4/6)	84.3 (5.6)	CDR = 0	4 (40%)	NR	0–II	IHC screening for LB, ubiquitin, p‐neurofilament and αB‐crystallin; not explicitly excluded or quantified
	ASYMAD	10 (5/5)	85.0 (6.3)	CDR = 0	3 (30%)	B	III–IV	
	AD	10 (6/4)	88.0 (5.8)	NR	4 (40%)	C	V–VI	
[39]	CT	13 (8/5)	84.8 (NR)	MMSE > 25	NR	C1^a^	I^a^	NR
	ASYMAD	10 (7/3)	89.5 (NR)	MMSE > 25	NR	C3^a^	V^a^	NR
	AD	21 (12/8)	81.0 (NR)	CDR > 1	NR	C3^a^	Vi^a^	NR
[40]	CT	10 (10/0)	86.2 (4.3)	MMSE > 26	0 (0%)	C0–1	I–III	Infarcts: 5; LB: 1
	ASYMad	10 (10/0)	84.8 (6.2)	MMSE > 26	7 (70%)	C2–3	III–V	Infarcts: 4; LB: 1
	AD	10 (10/0)	87.6 (4.4)	MMSE < 24	4 (40%)	C2–3	IV–V	Infarcts: 8; LB: 0
[41]	CT	15 (NR)	84.4 (3.2)	NR	NR	A	I–II	Excluded cases with significant non‐AD brain lesions
	ASYMAD (IP)	12 (NR)	89.8 (2.7)	NR	NR	B	III–IV	
	ASYMAD (HP)	15 (NR)	88.4 (6.0)	NR	NR	C	V–VI	
	AD	15 (NR)	87.2 (3.2)	NR	NR	C	V–VI	
[42]	CT	31 (16/15)	84.6 (4.3)	CDR = 0	NR	0–A	≤II	NR
	ASYMAD	12 (7/5)	85.3 (5.0)	CDR = 0	NR	B–C	≥IV	NR
	AD	19 (16/3)	86.1 (5.9)	CDR ≥ 2	NR	B–C	≥IV	NR
[43]	CT	15 (2/13)	84.0 (8.5)	NR	NR	0	0–IV	Excluded cases with significant non‐ad brain lesions
	ASYMAD	15 (3/12)	87.2 (8.1)	NR	NR	B–C	0–IV	
	MCI	15 (3/12)	87.0 (8.5)	NR	NR	A–B	0–IV	
	AD	15 (6/9)	92.0 (5.0)	NR	NR	B–C	II–VI	
[44]	CT	4 (3/1)	82.5 (7.0)	MMSE ≥ 29	NR	NR	I–II	NR
	ASYMAD	4 (3/1)	90.0 (0.0)	MMSE ≥ 26	NR	NR	IV–VI	NR
	MCI	3 (3/0)	89.7 (0.6)	MCI = 20–25	NR	NR	II–V	NR
	AD	6 (5/1)	81.0 (8.8)	MMSE ≤ 15	NR	NR	VI	NR
[45]	CT	14 (4/10)	82.6 (11.0)	MMSE ≥ 23	2 (14%)	NR	NR	NR
	ASYMAD	15 (5/10)	89.2 (7.9)	MMSE ≥ 25	4 (27%)	NR	NR	NR
	AD	14 (7/7)	87.9 (8.9)	MMSE ≤ 8	4 (29%)	NR	NR	NR
[46]	CT	8 (3/5)	92 (NR)	CDR < 1	NR	NR	I–III	NR
	ASYMad	8 (3/5)	90 (NR)	CDR < 1	NR	NR	IV–VI	NR
	AD	8 (3/5)	95 (NR)	CDR ≥ 1	NR	NR	IV	NR
[47]	CT	14 (4/10)	82.6 (11.0)	NR	2 (14.3%)	NR	NR	NR
	ASYMAD	15 (5/10)	89.2 (7.9)	NR	4 (26.7%)	NR	NR	NR
	AD	14 (7/7)	87.9 (8.9)	NR	4 (28.7%)	NR	NR	NR
[48]	CT	28 (10/18)	82.9 (11.6)	NR	NR	C0–2	I–II	Excluded cases with significant non‐AD brain lesions
	ASYMAD (IP)	21 (8/13)	86.6 (10.2)	NR	NR	C1–3	III–IV	
	ASYMAD (HP)	12 (7/5)	86.1 (10.1)	NR	NR	C2–3	V–VI	
	AD	29 (11/18)	83.1 (11.6)	NR	NR	C2–3	V–VI	
[49]	CT	14 (8/6)	94.7 (3.8)	CASI > 85	1 (7%)	0–C1	≤ III–IV	Microinfarcts 1.07; LB 1.00; LATE‐NC 0.21^b^
	ASYMad	7 (5/2)	85.2 (6.1)	CASI > 85	2 (29%)	C3	V–VI	Microinfarcts 0.57; LB 0.00; LATE‐NC 0.43^b^
	ad (matched to CT)	14 (8/6)	94.5 (2.5)	CASI ≤ 85	7 (50%)	C3	V–VI	Microinfarcts 1.50; LB 1.50; LATE‐NC 2.40^b^
	ad (matched to ASYMad)	7 (5/2)	86.7 (3.5)	CASI ≤ 85	4 (57%)	C3	V–VI	Microinfarcts 1.14; LB 0.57; LATE‐NC 1.86^b^
[50]	CT	13 (3/10)	82.4 (11.5)	NR	3 (23%)	≤ C1	NR	NR
	ASYMAD	13 (5/8)	88.2 (8.0)	NR	3 (23%)	> C1	NR	NR
	AD	17 (8/9)	87.4 (9.5)	NR	4 (23.5%)	> C1	NR	NR
[51]	CT	14 (10/4)	86.4 (14.8)	CDR = 0	NR	C0–1	0–III	NR
	ASYMAD	13 (8/5)	90.1 (4.0)	CDR = 0	NR	C2–3	II–VI	NR
	AD	16 (6/10)	83.2 (10.4)	CDR ≥ 1	NR	C2–3	V–VI	NR
[52]	CT	6 (5/1)	85.0 (7.4)	CDR < 1	NR	NR	I–IV	NR
	ASYMAD	5 (3/2)	89.9 (0.45)	CDR < 1	NR	NR	IV–VI	NR
	AD	4 (4/0)	78.3 (9.4)	CDR ≥ 1	NR	NR	III–VI	NR
[53]	CT	12 (9/3)	83.7 (3.5)	MMSE = 29–30	NR	NR	I–III	NR
	ASYMad	12 (6/6)	87.3 (3.5)	MMSE ≥ 27	NR	NR	III–VI	NR
	AD	10 (5/5)	83.6 (2.8)	MMSE < 10	NR	NR	IV–VI	NR
[54]	ASYMad	8 (7/1)	91.8 (5.2)	MMSE ≥ 19	NR	C1–3	IV–VI	NR
	AD	6 (5/1)	74.3 (9.5)	MMSE < 19	NR	C2–3	IV–VI	NR
[55]	CT	8 (4/4)	82.6 (8.3)	MMSE = 29^b^	0 (0%)	C0–2	0–II	Excluded cases with significant non‐ad brain lesions
	ASYMAD	20 (11/9)	88.9 (9.0)	MMSE = 29^b^	2 (10%)	C0–3	III–IV	
	AD	27 (13/14)	88.1 (6.1)	MMSE = 24^b^	4 (15%)	C0–3	III–IV	
[56]	CT	12 (7/5)	74‐100^d^	MMSE = 29–30	NR	NR	I–III	NR
	ASYMAD	12 (6/6)	82‐98^d^	MMSE ≥ 27	NR	NR	III–VI	NR
	AD	15 (8/7)	67‐103^d^	MMSE < 27	NR	NR	III–VI	NR
[57]	CT	24 (NR)	NR	MMSE > 23	NR	C0‐C1	0–III	NR
	ASYMAD	52 (NR)	NR	MMSE > 23	NR	C1‐C3	III–V	NR
	AD	32 (NR)	NR	MMSE < 24	NR	C2‐C3	III–VI	NR
[58]	CT	8 (4/4)	86.9 (7.4)	MMSE > 27	NR	C0	0–II	Excluded cases with significant non‐AD brain lesions
	ASYMAD	13 (7/6)	86.2 (10.6)	MMSE > 28	NR	C0–2	III–IV	
	AD	19 (8/11)	89.6 (6.3)	MMSE = 11–30	NR	C0–3	III–IV	
[59]	CT	6 (4/2)	95.7 (4.8)	MMSE = 29–30	NR	NR	II–III	NR
	ASYMAD	7 (3/4)	91.0 (4.9)	MMSE > 26	NR	NR	III–IV	NR
	AD	7 (2/5)	89.9 (4.3)	MMSE < 10	NR	NR	V–VI	NR
[60]	CT	9 (8/1)	85.2 (7.1)	MMSE = 29–30	NR	NR	I–II	NR
	ASYMAD	9 (5/4)	88.9 (6.5)	MMSE ≥ 26	NR	NR	IV–V	NR
	AD	9 (4/5)	80.6 (11.3)	MMSE ≤ 21	NR	NR	V–VI	NR
[61]	CT	13 (6/7)	76.8 (12.4)	CDR < 1	3 (23.1%)	0‐A	0–III	TDP43/LATE: 2
	ASYMAD	13 (7/6)	88.2 (5.5)	CDR < 1	2 (15.4%)	B‐C	IV–VI	TDP43/LATE: 4
	AD	19 (10/9)	82.7 (10.4)	CDR > 1	5 (26.3%)	B‐C	IV–VI	TDP43/LATE: 10
[62]	ASYMAD	4 (1/3)	91.5 (6.8)	MMSE ≥ 27	NR	NR	III–IV	NR
	AD	4 (1/3)	89.0 (5.2)	MMSE < 10	NR	NR	V–VI	NR
[63]	ASYMAD	4 (2/2)	90.8 (6.7)	MMSE > 26	NR	NR	III–IV	LB: 4; TDP‐43: 0
	AD	4 (2/2)	90.8 (3.3)	MMSE < 22	NR	NR	VI	LB: 3; TDP‐43: 0
	PART	4 (3/1)	85.8 (5.0)	MMSE > 25	NR	NR	I–II	LB: 4; TDP‐43: 0

*Note:* The table reports the main demographic and clinicopathological characteristics of the groups analysed in each study. Age is presented in years. Only clinical instruments for which quantitative group cut‐offs or summary scores were reported are listed. For the purpose of estimating age as a mean (SD), age values reported as ‘≥89’ were treated as 90 years.

Abbreviations: AD, Alzheimer's disease; APOE, apolipoprotein E; ASYMad, asymptomatic Alzheimer's disease; CASI, Cognitive Abilities Screening Instrument; CDR, Clinical Dementia Rating; CERAD, Consortium to Establish a Registry for Alzheimer's Disease; CT, cognitively unimpaired controls; F, female; HP, high pathology; IHC, immunohistochemistry; IP, intermediate pathology; LATE‐NC, limbic‐predominant age‐related TDP‐43 encephalopathy neuropathologic change; LB, Lewy bodies; M, male; MCI, mild cognitive impairment; MMSE, Mini‐Mental State Examination; NR, not reported; PART, primary age‐related tauopathy; TDP‐43, TAR DNA‐binding protein 43.

^a^ Data expressed as the median.

^b^ Data expressed as the mean.

^c^ Data expressed as allele frequency.

^d^ Data expressed as a range.

The mean age of ASYMad individuals ranged from 79.5 to 92.8 years. Sex distribution varied and included mixed‐sex samples, single‐sex cohorts and records in which sex was not reported. In the few studies that reported education, the mean duration of schooling in each group was at least 12 years. Reporting of race and ethnicity was scarce but suggested a marked predominance of White participants, accounting for more than 90% of the samples. The Clinical Dementia Rating and the Mini‐Mental State Examination were the most frequently used clinical instruments. These were often embedded within broader neurological assessments based on established diagnostic frameworks. All studies stated that neuropathological assessment followed CERAD and Braak criteria, consistent with our eligibility criteria. However, several reports did not provide the corresponding CERAD or Braak scores. Although some studies reported full APOE genotype distributions, others reported only ε4 carrier status or allele frequencies. Few studies provided measures of medical comorbidity, such as the Charlson Comorbidity Index, which was reported in one study. Most addressed comorbidity through exclusion criteria. Similarly, the assessment of copathologies varied widely, and a considerable number of records excluded major non‐ad brain lesions. A small subset of reports (17.6%) formally quantified and/or analysed mixed pathologies.

### 3.3 Risk of Bias

Risk of bias was assessed using the Newcastle–Ottawa Scale for case–control studies. Among the 34 studies, 17 (50.0%) achieved full scores in the Selection domain. Thirteen (38.2%) were downgraded by one point, and four (11.8%), by two points. Downgrades were mainly due to incompatibility in clinicopathological group definitions, for example, overlap in neuropathological severity across groups or insufficient reporting of group thresholds. In the Comparability domain, 13 studies (38.2%) controlled for age and at least one additional confounder, 17 (50.0%) controlled for age only, and four (11.8%) did not explicitly report matching or adjustment. All 34 records (100%) achieved full scores in the Exposure domain, reflecting standardised laboratory methods applied similarly across groups. Overall, 23 studies (67.6%) were classified as being at low risk of bias (8–9 stars) and 11 (32.4%) as being at moderate risk of bias (5–7 stars). The mean Newcastle–Ottawa Scale score was 7.65 ± 0.92. The complete risk of bias assessment for each study is presented in Table S1.

### 3.4 Proteinopathy Profiles

ASYMad differed from symptomatic ad in the regional burden, biochemical forms and subcellular localisation of Aβ and p‐tau. Reduced Aβ accumulation was identified in the entorhinal cortex, hippocampal CA1, superior frontal gyrus, midbrain and cerebellum of ASYMad individuals [41, 43, 49]. Reduced tau pathology was observed in cingulate regions, hippocampal subfields (CA1–CA4, subiculum and dentate gyrus) and the middle frontal gyrus [43, 49, 51]. One study also reported reduced vascular amyloid in ASYMad, together with higher levels of amyloid‐processing proteins [37]. In terms of morphology, ASYMad showed a higher proportion of compact plaques and smaller fibrillar plaques [41, 61]. Multiple studies supported reduced accumulation of soluble Aβ and p‐tau oligomers in ASYMad [30, 39, 41, 51, 59]. Notably, several of these studies also reported reduced synaptic accumulation of oligomeric species in this group [39, 41, 51, 58, 59].

Additional evidence highlighted qualitative differences in protein assemblies. Marcatti et al. [62] reported greater structural stability and lower cytotoxic and synaptotoxic potential of tau oligomers in ASYMad, whereas Jamison et al. [63] found tau interactomes enriched for cellular antioxidant responses. Tumurbaatar et al. [59] linked alterations in proteinopathy to differences in clearance pathways, suggesting preserved autophagy and mitophagy in ASYMad, as well as inverse associations between autophagic proteins and tau oligomers. Corroborating evidence from Jury‐Garfe et al. [61] further indicated lower p‐tau seeding activity and upregulation of endocytosis, autophagy and phagocytosis in this group. Notably, Latimer et al. [49] reported a lower burden of mixed pathologies in ASYMad brains, particularly LATE‐NC and cerebrovascular lesions. However, differences in copathologies were not reported in most studies.

### 3.5 Neuronal and Synaptic Integrity

Multiple studies indicated preserved cellular integrity in ASYMad compared with symptomatic ad. Studies reported greater neuronal number and cortical thickness in the entorhinal cortex, hippocampal CA1 and superior temporal sulcus in ASYMad [31, 32, 41]. Latimer et al. [49] further reported less cortical microvacuolar injury in ASYMad than in symptomatic ad, most prominently in the temporal cortex. In addition, one study analysing the hippocampus found lower levels of markers of neuronal cell death in ASYMad, as assessed by TUNEL [42]. Morphometric analyses also described increased volumes of neuronal cell bodies, nuclei and nucleoli across the hippocampus (CA1), cingulate regions and primary visual cortex in ASYMad brains [33, 34, 35].

One study analysing frontal cortex tissue reported less rarefaction of white matter in ASYMad than in symptomatic ad [37]. Additional axonal measures supported less axonal distortion both near and distant from plaques in ASYMad than in symptomatic ad [41]. Guptarak et al. [60] assessed dendritic spines and reported greater spine density in the frontal cortex of ASYMad individuals, particularly long, thin filopodial spines. The same study also reported higher Pin1 levels, with a more balanced distribution between neuronal processes and the soma [60].

Fracassi et al. [53] assessed oxidative stress and mitochondrial responses and reported lower oxidative damage, measured by 8‐oxo‐dG and 4‐HNE, in neurons and astrocytes of ASYMad brains. They also found upregulation of antioxidant response pathways, including PGC1α/PPARα, together with lower miR‐485 expression, compared with symptomatic ad [53]. Similarly, Walker et al. [54] found lower expression of stress‐related, oxidative and metabolic markers, including PINK1 and IDH1, in the hippocampus of individuals with ASYMad.

Furthermore, several studies converged on synaptic preservation as a key feature distinguishing ASYMad from symptomatic ad. One study reported greater synaptic density in the entorhinal cortex and superior frontal gyrus in ASYMad [30]. Analysis of the visual cortex showed greater densities of presynaptic and postsynaptic puncta in ASYMad, together with reduced glial engulfment of synaptic elements [58]. Similarly, Arnold et al. [40] reported higher synaptophysin expression and a greater number of synaptopodin‐positive spines in the midfrontal cortex of this group than in symptomatic ad. Corroborating evidence from Singh et al. [51] indicated preserved synaptic functional integrity in ASYMad, including preserved electrophysiological glutamatergic and GABAergic receptor function associated with synaptic plasticity, particularly long‐term potentiation.

At the molecular level, Bjorklund et al. [39] reported increased expression of phosphorylated CREB and preserved zinc homeostasis in the postsynaptic fractions of ASYMad brains compared with symptomatic ad. Zolochevska et al. [46] identified a distinct plasticity‐associated proteomic signature in the postsynaptic density of ASYMad, mainly characterised by increased expression of ANXA2 and CAMK2A. A subsequent analysis by the same authors suggested that the microRNAs miR‐4723, miR‐149 and miR‐485 were key drivers of the proteomic differences observed between groups [52]. Similarly, Hurst et al. [57] reported preserved synapse‐related proteomic signatures in ASYMad, with higher abundance of NRN1. This neurotrophic factor was also identified as a hub protein within resilience‐associated modules [57].

Complementary evidence from Briley et al. [44] suggested enhanced neurogenesis, indicated by SOX2+/NeuN+, in the dentate gyrus of ASYMad brains compared with symptomatic ad, alongside lower expression of miR‐25 and miR‐124. A transcriptomic study further reported alterations in the expression of neuronal genes related to learning and memory in multiple regions of ASYMad brains that were not observed in symptomatic ad [36]. Finally, one hippocampal study reported reduced expression of markers involved in cell cycle progression in ASYMad, alongside improved activity of DNA repair signatures [42].

### 3.6 Brain Metabolism

Distinct metabolic signatures were reported when comparing ASYMad and symptomatic ad brains. Snowden et al. [45] found a preserved profile of polyunsaturated fatty acids (PUFAs) in the middle frontal gyrus of ASYMad brains, with higher levels of eicosapentaenoic, linolenic, linoleic, oleic and arachidonic acids and lower levels of docosahexaenoic acid than in symptomatic ad. Mahajan et al. [50] reported differences in metabolites linked to transmethylation and polyamine pathways, including higher levels of choline, N‐acetylglutamate, N‐acetylaspartate, gamma‐aminobutyric acid and glutathione and lower levels of S‐adenosylhomocysteine, S‐adenosylmethionine, cysteine and spermidine in ASYMad. Complementary evidence from An et al. [47] indicated that glucose concentration in brain tissue from the inferior temporal gyrus followed the pattern AD > ASYMAD > controls, whereas glycolytic enzyme activity followed the pattern AD < ASYMAD < controls.

### 3.7 Glial and Immune Activity

The evidence synthesised in this review suggests differences in glial and immune activity in ASYMad compared with symptomatic ad. In the frontal cortex, Fracassi et al. [56] reported higher microglial immunoreactivity (IBA1) around Aβ plaques, together with increased expression of markers consistent with efficient engulfment of debris and damaged synapses (CD68, TREM2/DAP12 and PSD95). Jury‐Garfe et al. [61] similarly reported increased numbers of microglia surrounding filamentous plaques, higher expression of actin‐related proteins involved in motility and phagocytosis (ARP2 and CAP1) and increased activity of pathways related to phagocytosis, endocytosis and autophagy in the middle frontal gyrus of ASYMad individuals compared with symptomatic AD. Arnold et al. [40] further identified higher densities of GFAP+ astrocytes in the midfrontal gyrus cortex of ASYMAD.

In contrast, three studies reported lower glial activity in the entorhinal cortex, hippocampus and superior temporal sulcus of ASYMAD than of symptomatic AD, including fewer GFAP+ astrocytes and lower CD68 immunoreactivity [41, 48, 54, 55]. Corroborating evidence from Taddei et al. [55] suggested higher expression of homeostatic microglial markers (TMEM119+ and P2RY12+) in the temporal lobe of ASYMad. Beyond cellular markers, Morimoto et al. [38] assessed transcripts of inflammatory mediators in the middle temporal cortex and reported lower mRNA expression of IL‐1β, IL‐10, IL‐13, IL‐18, IL‐33, TACE and TGFβ1 in ASYMAD than in symptomatic AD. Similarly, Barroeta‐Espar et al. [48] described cytokine patterns combining increases in selected cytokines with decreases in chemokines linked to immune recruitment, such as MCP‐1 and MIP‐1α, in the entorhinal cortex and superior temporal sulcus of ASYMAD brains. Walker et al. [54] reported lower expression of several inflammatory markers in the hippocampus of this group than in symptomatic AD.

### 3.8 Summary of the Main Findings

Table 2 summarises the main findings of the studies included in this review. The mean post‐mortem interval ranged from 2.02 to 27.1 h. Brain regions analysed included both regions classically vulnerable to AD pathology, such as the entorhinal cortex, hippocampus and frontal cortex, and less vulnerable regions, such as the visual cortex, brainstem and cerebellum [64]. A wide range of analytical approaches was used. The most common included (i) histological and immunolabelling methods, such as immunohistochemistry, immunofluorescence, stereology and morphometry; (ii) protein quantification platforms, such as ELISA, western blotting, microarrays, two‐dimensional gel electrophoresis and mass spectrometry; (iii) gene expression assays, such as RNA sequencing and qRT‐PCR or real‐time PCR; and (iv) cytometric and chromatographic platforms, such as flow cytometry and metabolomics or lipidomics workflows using capillary electrophoresis time‐of‐flight MS or gas chromatography‐MS.

**TABLE 2:** Summary of the main post‐mortem brain tissue findings that differ between symptomatic and asymptomatic Alzheimer's disease.

REF	Brain region	Main findings (ASYMAD vs. ad)
[30]	Entorhinal cortex and superior frontal gyrus	↑ synaptic density (synaptophysin) ↓ soluble Aβ_40_ and Aβ_42_
[31]	Entorhinal cortex and hippocampus (CA1)	↑ neuronal number and volume
[32]	Hippocampus	↑ neuronal number in CA1
[33]	Anterior cingulate gyrus and hippocampus (CA1)	↓ neuronal soma atrophy ↑ neuronal nuclear volume
[34]	Hippocampus (CA1), anterior cingulate gyrus, posterior cingulate gyrus and primary visual cortex	↑ volume of neuronal cell bodies, nuclei and nucleoli
[35]	Hippocampus (CA1)	↑ volume of neuronal cell bodies, nuclei and nucleoli
[36]	Entorhinal cortex, hippocampus, middle temporal gyrus, posterior cingulate cortex, superior frontal gyrus and primary visual cortex	Altered neuronal gene expression in learning‐ and memory‐related pathways compared with controls, a pattern not observed in ad: ↓ APOE, EGR1, FYN, PTN, S100B ↑ EFNB2, GRM7, LAMB1, PRKACB and VDAC3
[37]	Frontal lobe	↓ vascular amyloid (total cerebral amyloid angiopathy) ↓ white matter rarefaction ↑ Aβ_42_, CD200, soluble ApoE and APP holoprotein BACE‐1 VEGF ↓ IDE 110, PEDF and pro‐BDNF
[38]	Middle temporal cortex and cerebellum	↓ mRNA expression of IL‐1β, IL‐10, IL‐13, IL‐18, IL‐33, TACE and TGFβ1
[39]	Mid‐hippocampus	↓ Aβ oligomers in the postsynaptic fractions ↑ phosphorylated CREB ↓ Zn^2+^ and ↑ ZnT3
[40]	Midfrontal gyrus cortex (BA46)	↑ GFAP+ astrocyte density ↑ synaptopodin‐positive spines and synaptophysin ↑ APP trafficking/APP interaction (APPBP1), nuclear export (XPO2) ↓ cell cycle markers (Cyclin D3, MCM5), apoptosis receptors (Fas/TNFRSF6)
[41]	Superior Temporal Sulcus	↓ fibrillar Aβ plaques (10D5+) ↓ oligomeric Aβ (NAB61+) ↑ neuronal number and cortical thickness ↓ glial activity (CD68+, GFAP+) ↓ synaptic soluble tau oligomers and ↑ cytosolic tau monomers
[42]	Hippocampus	↓ cell cycle progression markers (Cdk4, cyclin D, phospho‐Rb, E2F1, Cdk1, cyclin B) ↓ neuronal cell death (TUNEL) ↑ DNA‐repair and cell cycle inhibitory signatures (nuclear p53, p27, BRCA1, PTEN, Cdk5)
[43]	Anterior cingulate gyrus, posterior cingulate gyrus and hippocampus (CA1)	↓ diffuse‐Aβ in CA1 ↓ tau‐NFTs in the anterior cingulate gyrus and posterior cingulate gyrus ↓ neuropil tau threads in the anterior cingulate gyrus
[44]	Hippocampus	↓ miR‐25 and miR‐124 in the dentate gyrus ↑ neurogenesis markers (SOX2+/NeuN+)
[45]	Cerebellum, middle frontal gyrus and inferior temporal gyrus	↑ EPA, linolenic, linoleic, oleic and arachidonic acid in the middle frontal gyrus ↓ DHA in the middle frontal gyrus
[46]	Mid‐hippocampus	↑ ANXA2 and CAMK2A in the postsynaptic proteome
[47]	Inferior temporal gyrus, middle frontal gyrus and cerebellum	↓ brain tissue glucose in the inferior temporal gyrus (ad > ASYMAD > CT) ↑ glycolytic enzyme activity for HK‐PFK and PK in the inferior temporal gyrus (AD < ASYMAD < CT)
[48]	Entorhinal cortex and superior temporal sulcus	↑ neuronal count and cortical thickness ↓ GFAP+ astrocytes and CD68 + microglia ↑ IL‐1β, IL‐6, IL‐10, IL‐1ra, IL‐13 and IL‐4 ↓ IL‐17, RANTES, MIP‐1β, TNF‐α, IL‐5, Eotaxin, MIP‐1α ↑ neurotrophic factors (↑ PDGF‐bb; ↑ basic FGF) ↓ chemokines linked to microglial recruitment (↓ MCP‐1, ↓ MIP‐1α)
[49]	Cerebral cortex, brainstem and cerebellum	↓ Aβ in the midbrain and the cerebellum ↓p‐tau in the middle frontal gyrus ↑ synaptic integrity within the perforant pathway ↓ cortical microvacuolar change, particularly in the temporal cortex ↓ pTDP‐43/LATE in the hippocampus, entorhinal cortex and transentorhinal cortex ↓ macroscopic infarcts
[50]	Cerebellum, middle frontal gyrus and inferior temporal gyrus	↑ choline, NAG, NAA, GABA and GSH ↓ s‐adenosylhomocysteine, s‐adenosylmethionine, cysteine and spermidine
[51]	Hippocampus and frontal cortex	↓ total and oligomeric tau ↓ synaptic tau oligomers (Tau5/T22, HT7/PSD‐95) ↑ synaptic functional integrity (functional state of glutamate and GABA receptors; microtransplantation electrophysiology; FASS‐LTP)
[52]	Hippocampus and frontal cortex	Similar levels of miR‐149, miR‐485 and miR‐4723 in ASYMad and controls ↑ miR‐149 and miR‐485 and ↓ miR‐4723 levels in ad versus controls
[53]	Frontal cortex	↓ oxidative damage (8‐oxo‐dG; 4‐HNE) in neurons and astrocytes ↑ antioxidant mitochondrial response (SOD2) Improved regulation of antioxidant response (e.g., ↑ PGC1α/PPARα and ↓ miR‐485)
[54]	Hippocampus	↓ proteinopathy and protein‐processing signals (↓ APP, ↓ neprilysin, ↓ IDE, ↓ p‐tau S396/S404, ↓ ubiquitin) ↑ markers of synaptic and neuronal integrity (SYP, PSD‐95 and NEFL) ↓ inflammatory and reactive glial markers (CD68, GFAP and CD39) ↓ stress‐related and oxidative‐metabolic markers (PINK1 and IDH1)
[55]	Temporal lobe and visual cortex	↑ homeostatic microglia (TMEM119+ and P2RY12+) ↓ astrocytic (GFAP+) and microglial activity (CD68+ and HLA‐DR+)
[56]	Frontal cortex	↑ reactive microglia around plaques (IBA1+, TREM2+, DAP12+ and CD68+) ↑ microglia engulfment of debris and damaged synapses (PSD95+ and CD68) ↑ preservation of synapses around plaques (MAP 2+ and PSD95+)
[57]	Frontal cortex (BA6) and temporal cortex (BA37)	Preserved synapse‐related proteomic signatures ↑ NRN1
[58]	Visual cortex	↑ synapse density (synapsin‐1 presynaptic and PSD95 postsynaptic puncta) ↓ DNA damage/stress (γH2AX) ↓ microglial and astrocytic engulfment of synaptic elements ↓ synaptic tau pathology, including p‐tau (Thr181) and tau oligomers (TOC1+)
[59]	Hippocampus	↓ oligomeric p‐tau accumulation ↑ autophagy and mitophagy‐related proteins
[60]	Frontal cortex	↑ spine density and ↓ spine dimensions ↑ density of long thin and filopodia spines ↓ density of mushroom spines ↑ Pin1 near plaques ↑ distribution of Pin1 between cell processes and soma
[61]	Middle frontal gyrus	↓ filamentous Aβ plaques ↓ p‐tau seeding activity ↑ microglia surrounding filamentous plaques ↑ actin‐based microglia motility proteins ↑ activity of autophagy‐, endocytosis‐ and phagocytosis‐related pathways
[62]	Hippocampus	Tau oligomers polymorphs exhibited: ↑ size (diameter and height) ↑ resistance to proteolysis ↓ cytotoxic and synaptotoxic potential
[63]	Hippocampus	↑ proteins involved in cellular responses to reactive oxygen species in tau oligomers interactomes

*Note:* The table summarises the main post‐mortem brain tissue findings reported across studies comparing ASYMad and symptomatic ad. Arrows indicate the direction of change in ASYMad relative to AD (↑ higher or increased; ↓ lower or decreased).

Abbreviations: 4‐HNE, 4‐hydroxynonenal; 8‐oxo‐dG, 8‐oxo‐2′‐deoxyguanosine; Aβ, amyloid‐beta; AD, Alzheimer's disease; APP, amyloid precursor protein; ApoE, apolipoprotein E; ASYMAD, asymptomatic Alzheimer's disease; BA, Brodmann area; BACE‐1, β‐site APP‐cleaving enzyme 1; BRCA1, breast cancer type 1 susceptibility protein; CAA, cerebral amyloid angiopathy; Cdk, cyclin‐dependent kinase; CD68, cluster of differentiation 68; CREB, cAMP response element‐binding protein; DAP12, DNAX‐activation protein 12; DHA, docosahexaenoic acid; EPA, eicosapentaenoic acid; FASS‐LTP, fluorescence analysis of synaptosome‐associated proteins and long‐term potentiation; GFAP, glial fibrillary acidic protein; GABA, gamma‐aminobutyric acid; GSH, glutathione; HLA‐DR, human leukocyte antigen‐DR; IBA1, ionised calcium‐binding adaptor molecule 1; IDH1, isocitrate dehydrogenase 1; IDE, insulin‐degrading enzyme; IL, interleukin; LATE, limbic‐predominant age‐related TDP‐43 encephalopathy; MAP 2, microtubule‐associated protein 2; MCP‐1, monocyte chemoattractant protein 1; miR, microRNA; MIP‐1α, macrophage inflammatory protein 1 alpha; MIP‐1β, macrophage inflammatory protein 1 beta; mRNA, messenger RNA; NAA, N‐acetylaspartate; NAG, N‐acetylglutamate; NEFL, neurofilament light chain; NeuN, neuronal nuclei; NFT, neurofibrillary tangle(s); NRN1, neuritin 1; pCREB, phosphorylated CREB; PDGF‐bb, platelet‐derived growth factor BB; phospho‐Rb, phosphorylated retinoblastoma protein; PINK1, PTEN‐induced kinase 1; PSD95, postsynaptic density protein 95; PTEN, phosphatase and tensin homologue; p‐tau, phosphorylated tau; P2RY12, purinergic receptor P2Y12; RANTES, regulated upon activation, normal T cell expressed and secreted; SOX2, sex‐determining region Y‐box 2; SOD2, superoxide dismutase 2; synapsin‐1, synapsin‐1; SYP, synaptophysin; TACE, TNF‐α‐converting enzyme; TGFβ1, transforming growth factor beta 1; TMEM119, transmembrane protein 119; TNF‐α, tumour necrosis factor alpha; TREM2, triggering receptor expressed on myeloid cells 2; TUNEL, terminal deoxynucleotidyl transferase dUTP nick‐end labelling; VEGF, vascular endothelial growth factor; Zn^2+^, zinc ion; ZnT3, zinc transporter 3.

## 4 Discussion

In this systematic review, we aimed to identify post‐mortem brain tissue findings that differ between symptomatic and asymptomatic ad. Overall, the evidence clustered into four domains: (i) proteinopathy profiles, (ii) neuronal and synaptic integrity, (iii) metabolic pathways and (iv) glial and immune signatures. Lower levels of oligomeric Aβ and p‐tau were observed in ASYMad, alongside reduced synaptic accumulation of these species [30, 39, 41, 51, 58, 59]. Notably, soluble oligomers are frequently implicated as key drivers of synaptotoxicity and cognitive decline in ad [65, 66, 67, 68, 69, 70]. Differences related to oligomers were accompanied by preserved activity of clearance pathways, such as autophagy, and antioxidant responses to proteinopathy in ASYMAD [42, 53, 62, 63, 71, 72, 73]. These findings support the presence of a distinct molecular profile, subcellular compartmentalisation and cellular handling of toxic proteins in ASYMAD compared with symptomatic AD, despite a similar overall burden of plaques and tangles [41]. It is plausible that these differences contribute to relatively less damage to the neuronal and synaptic environment [15].

Corroborating this hypothesis, multiple neuronal and synaptic findings in ASYMad suggested reduced damage and preserved function. The evidence in this area was broad and included reduced tissue injury, increased neurogenesis, neuronal hypertrophy, plasticity‐related synaptic proteomic and functional signatures and preservation of axonal and dendritic structures [31, 32, 33, 34, 35, 36, 40, 42, 44, 46, 51, 52, 54, 57, 60]. Some studies also suggested partial preservation of metabolic homeostasis, including PUFA, one‐carbon and polyamine and glucose metabolism pathways previously found to be disrupted in symptomatic ad [74, 75, 76, 77, 78, 79, 80, 81]. However, the breadth and descriptive nature of these findings indicate that the maintenance of neuronal health in resilient individuals is unlikely to be explained by a single marker or pathway. The underlying mechanisms remain to be elucidated in future research.

In addition to neuronal differences, there were also meaningful distinctions in glial and immune activity, suggesting a context‐dependent activation profile. Interestingly, ASYMad showed greater astrocytic reactivity (GFAP) and increased plaque‐associated microglial engagement and phagocytic activity in frontal regions, for example, TREM2/DAP12, CD68, CAP1 and ARP2 [40, 56, 61, 82, 83, 84, 85, 86]. In contrast, temporal and entorhinal regions in ASYMad exhibited lower glial reactivity and preserved homeostatic microglial signatures, such as TMEM119 and P2RY12, alongside reduced cytokine expression [38, 41, 48, 55, 87, 88, 89]. A parsimonious interpretation is that this reflects a balance between effective local responses to pathology and maintenance of homeostatic function within vulnerable networks affected early in ASYMad [7, 64]. This could hypothetically limit exposure to prolonged, non‐resolving neuroinflammatory activity, a feature commonly implicated in the progression of neurodegenerative diseases [90, 91, 92]. However, this interpretation should be regarded as hypothesis‐generating, as the included studies were not designed to test immune mechanisms or longitudinal change.

Although this review identifies multiple biological processes that may be associated with the ASYMad phenotype, several limitations should be acknowledged. First, most studies were conducted in a single high‐income country, the United States, whereas the greatest burden of ad lies in low‐ and middle‐income countries, thereby limiting generalisability [93]. Reporting of key metadata, such as age, sex, education, ethnicity, neuropathological staging, genotyping and post‐mortem interval, was also frequently incomplete. Notably, the brain regions analysed varied considerably, and several effects appeared to be region‐dependent [94, 95, 96].

In addition, the pathological definitions of control, ASYMad and symptomatic ad groups were not standardised, and overlap in CERAD and Braak classifications across groups was common. This is particularly relevant because the concept of ASYMAD presupposes ADNC comparable to that of symptomatic AD but in the absence of clinical dementia [15, 16, 97]. Without adequate matching for pathology stage and brain region, the reported differences may partly reflect an earlier stage of disease rather than mechanisms of resilience to AD pathology per se [98]. Another major limitation is that mixed neuropathologies, including Lewy body disease, TDP‐43/LATE‐NC and cerebrovascular lesions, were not systematically assessed; only one study reported a lower burden of mixed pathologies in ASYMAD [49]. Given that ‘pure’ AD accounts for only a minority of cases, approximately 26%, unmeasured copathologies or their exclusion, substantially limit the generalisability of the findings [99, 100, 101, 102, 103].

## 5 Future Directions for Post‐Mortem Studies

A key implication of this review is the need for harmonised protocols and minimum reporting standards in research on the ASYMAD phenotype. We propose that future studies should prioritise: (i) standardised ADNC criteria and explicit thresholds, with careful pathology stage matching between ASYMAD and symptomatic AD groups; (ii) systematic assessment and reporting of major copathologies; and (iii) complete reporting of essential metadata, such as age, sex, education, race and ethnicity, comorbidities, genotypes, post‐mortem interval and power or sample size considerations. Based on the 2012 NIA‐AA recommendations for the neuropathological diagnosis of AD [11], future studies should, where feasible, explicitly report pathology staging for ASYMAD individuals using the ABC score (A = Thal Aβ phase, B = Braak NFT stage and C = CERAD neuritic plaque score), together with the corresponding level of ADNC (not, low, intermediate or high). A pragmatic minimum criterion for allocation to the ASYMAD group could be at least Intermediate ADNC, as defined by the NIA‐AA ABC scheme [11]. Importantly, even with such standardisation, careful pathology‐stage matching between ASYMAD and symptomatic AD groups will remain essential to increase confidence that reported differences reflect resilience mechanisms rather than disease stage effects [98].

## 6 Conclusion

In conclusion, beyond the limitations outlined above, this review suggests that ASYMAD brains may exhibit selected neuropathological and molecular features that differ from those of symptomatic AD. The exploratory differences identified in proteinopathy profiles, neuronal integrity, metabolic pathways and glial signatures warrant further investigation in mechanistic studies. Clarifying and validating these findings could ultimately contribute to the identification of targets aimed at delaying or reducing AD symptoms.

## Author Contributions

**Thiago Guilherme Rêgo Barros:** conceptualisation, writing – original draft, investigation, methodology. **Laura Barbosa Grossi:** conceptualisation, writing – original draft, investigation, methodology. **Maria Letícia da Silva Campos:** writing – original draft. **Israel Bem‐Hur Netto Cardoso:** validation, visualisation. **Antônio Lúcio Teixeira:** writing – review and editing, supervision. **Aline Silva de Miranda:** writing – review and editing, supervision. **Eliana Cristina de Brito Toscano:** conceptualisation, writing – review and editing, supervision, project administration.

## Funding

The authors have received financial support from the Brazilian government funding agencies: Fundação de Amparo à Pesquisa do Estado de Minas Gerais—FAPEMIG (APQ 0158424), Conselho Nacional de Desenvolvimento Científico e Tecnológico—CNPq (306078/2025‐2) and Coordenação de Aperfeiçoamento de Pessoal de Nível Superior (CAPES).

## Ethics Statement

This work was based on published literature, thereby not requiring direct institutional ethical approval.

## Conflicts of Interest

The authors declare no conflicts of interest.

## Supporting information


**Table S1:** Newcastle–Ottawa Scale risk of bias assessment.

## Data Availability

No datasets were generated during this research. All data supporting the findings are contained within the manuscript and derived from the cited studies. The protocol for this review is registered in PROSPERO (CRD42024568239).
