# Supplementary material for: Asymptomatic Versus Symptomatic Alzheimer's Disease Neuropathology: A Systematic Review of Differences Reported in Post‐Mortem Studies
Source: Neuropathol Appl Neurobiol. 2026 May 8;52:e70077. doi: 10.1111/nan.70077 (PMC13156076; doi:10.1111/nan.70077)
Supplement: Supplementary file 1 — Table S1: Newcastle–Ottawa Scale risk of bias assessment. [file NAN-52-e70077-s001.docx]

**Supplementary Table 1 - Newcastle-Ottawa Scale risk of bias assessment**

| **REF** | **Selection** | **Comparability** | **Exposure** | **Total** |
| --- | --- | --- | --- | --- |
| [1] | 3 | 2 | 3 | 8 |
| [2] | 3 | 0 | 3 | 6 |
| [3] | 3 | 1 | 3 | 7 |
| [4] | 3 | 1 | 3 | 7 |
| [5] | 3 | 1 | 3 | 7 |
| [6] | 4 | 1 | 3 | 8 |
| [7] | 2 | 0 | 3 | 5 |
| [8] | 4 | 1 | 3 | 8 |
| [9] | 3 | 2 | 3 | 8 |
| [10] | 4 | 1 | 3 | 8 |
| [11] | 3 | 2 | 3 | 8 |
| [12] | 4 | 1 | 3 | 8 |
| [13] | 4 | 2 | 3 | 9 |
| [14] | 3 | 2 | 3 | 8 |
| [15] | 4 | 1 | 3 | 8 |
| [16] | 2 | 2 | 3 | 7 |
| [17] | 4 | 1 | 3 | 8 |
| [18] | 2 | 2 | 3 | 7 |
| [19] | 4 | 1 | 3 | 8 |
| [20] | 4 | 2 | 3 | 9 |
| [21] | 4 | 2 | 3 | 9 |
| [22] | 3 | 1 | 3 | 7 |
| [23] | 3 | 0 | 3 | 6 |
| [24] | 4 | 1 | 3 | 8 |
| [25] | 3 | 0 | 3 | 6 |
| [26] | 2 | 2 | 3 | 7 |
| [27] | 4 | 1 | 3 | 8 |
| [28] | 4 | 2 | 3 | 9 |
| [29] | 3 | 2 | 3 | 8 |
| [30] | 4 | 1 | 3 | 8 |
| [31] | 4 | 1 | 3 | 8 |
| [32] | 4 | 1 | 3 | 8 |
| [33] | 4 | 1 | 3 | 8 |
| [34] | 3 | 2 | 3 | 8 |

Caption: Risk of bias assessments using the Newcastle-Ottawa Scale for case-control studies. Stars for the Selection domain were awarded when group assignment was based on adequate clinical and neuropathological thresholds. In the Comparability domain, one star was assigned when age was matched, and a second star when at least one additional confounder was adjusted for, for example sex, education, APOE, or PMI. The Exposure domain was rated as low risk of bias when neuropathological and laboratory measures were ascertained using standardised methods across groups. Abbreviations: APOE, apolipoprotein E; PMI, post-mortem interval.

**REFERENCES**

1. Lue LF, Kuo YM, Roher AE, et al. Soluble Amyloid β Peptide Concentration as a Predictor of Synaptic Change in Alzheimer’s Disease. *Am J Pathol*. 1999;155(3):853-862. doi:10.1016/S0002-9440(10)65184-X

2. Price JL, Ko AI, Wade MJ, Tsou SK, McKeel DW, Morris JC. Neuron Number in the Entorhinal Cortex and CA1 in Preclinical Alzheimer Disease. *Arch Neurol*. 2001;58(9):1395-1402. doi:10.1001/archneur.58.9.1395

3. West MJ, Kawas CH, Stewart WF, Rudow GL, Troncoso JC. Hippocampal neurons in pre-clinical Alzheimer’s disease. *Neurobiol Aging*. 2004;25(9):1205-1212. doi:10.1016/j.neurobiolaging.2003.12.005

4. Riudavets MA, Iacono D, Resnick SM, et al. Resistance to Alzheimer’s pathology is associated with nuclear hypertrophy in neurons. *Neurobiol Aging*. 2007;28(10):1484-1492. doi:10.1016/j.neurobiolaging.2007.05.005

5. Iacono D, O’Brien R, Resnick SM, et al. Neuronal Hypertrophy in Asymptomatic Alzheimer Disease. *J Neuropathol Exp Neurol*. 2008;67(6):578-589. doi:10.1097/NEN.0b013e3181772794

6. Iacono D, Markesbery WR, Gross M, et al. The Nun Study. *Neurology*. 2009;73(9):665-673. doi:10.1212/WNL.0b013e3181b01077

7. Liang WS, Dunckley T, Beach TG, et al. Neuronal gene expression in non-demented individuals with intermediate Alzheimer’s Disease neuropathology. *Neurobiol Aging*. 2010;31(4):549-566. doi:10.1016/j.neurobiolaging.2008.05.013

8. Maarouf CL, Daugs ID, Kokjohn TA, et al. Alzheimer’s Disease and Non-Demented High Pathology Control Nonagenarians: Comparing and Contrasting the Biochemistry of Cognitively Successful Aging. *PLoS ONE*. 2011;6(11):e27291. doi:10.1371/journal.pone.0027291

9. Morimoto K, Horio J, Satoh H, et al. Expression Profiles of Cytokines in the Brains of Alzheimer’s Disease (AD) Patients Compared to the Brains of Non-Demented Patients with and without Increasing AD Pathology. *J Alzheimer’s Dis*. 2011;25(1):59-76. doi:10.3233/JAD-2011-101815

10. Bjorklund NL, Reese LC, Sadagoparamanujam VM, Ghirardi V, Woltjer RL, Taglialatela G. Absence of amyloid β oligomers at the postsynapse and regulated synaptic Zn2+ in cognitively intact aged individuals with Alzheimer’s disease neuropathology. *Mol Neurodegener*. 2012;7:23. doi:10.1186/1750-1326-7-23

11. Arnold SE, Louneva N, Cao K, et al. Cellular, synaptic, and biochemical features of resilient cognition in Alzheimer’s disease. *Neurobiol Aging*. 2013;34(1):157-168. doi:10.1016/j.neurobiolaging.2012.03.004

12. Perez-Nievas BG, Stein TD, Tai HC, et al. Dissecting phenotypic traits linked to human resilience to Alzheimer’s pathology. *Brain J Neurol*. 2013;136(Pt 8):2510-2526. doi:10.1093/brain/awt171

13. Silva ART, Santos ACF, Farfel JM, et al. Repair of Oxidative DNA Damage, Cell-Cycle Regulation and Neuronal Death May Influence the Clinical Manifestation of Alzheimer’s Disease. *PLOS ONE*. 2014;9(6):e99897. doi:10.1371/journal.pone.0099897

14. Iacono D, Resnick SM, O’Brien R, et al. Mild cognitive impairment and asymptomatic Alzheimer disease subjects: equivalent β-amyloid and tau loads with divergent cognitive outcomes. *J Neuropathol Exp Neurol*. 2014;73(4):295-304. doi:10.1097/NEN.0000000000000052

15. Briley D, Ghirardi V, Woltjer R, et al. Preserved neurogenesis in non-demented individuals with AD neuropathology. *Sci Rep*. 2016;6(1):27812. doi:10.1038/srep27812

16. Snowden SG, Ebshiana AA, Hye A, et al. Association between fatty acid metabolism in the brain and Alzheimer disease neuropathology and cognitive performance: A nontargeted metabolomic study. *PLoS Med*. 2017;14(3):e1002266. doi:10.1371/journal.pmed.1002266

17. Zolochevska O, Bjorklund N, Woltjer R, Wiktorowicz JE, Taglialatela G. Postsynaptic Proteome of Non-Demented Individuals with Alzheimer’s Disease Neuropathology. Puzzo D, ed. *J Alzheimer’s Dis*. 2018;65(2):659-682. doi:10.3233/JAD-180179

18. An Y, Varma VR, Varma S, et al. Evidence for brain glucose dysregulation in Alzheimer’s disease. *Alzheimers Dement*. 2018;14(3):318-329. doi:10.1016/j.jalz.2017.09.011

19. Barroeta-Espar I, Weinstock LD, Perez-Nievas BG, et al. Distinct cytokine profiles in human brains resilient to Alzheimer’s pathology. *Neurobiol Dis*. 2019;121:327-337. doi:10.1016/j.nbd.2018.10.009

20. Latimer CS, Burke BT, Liachko NF, et al. Resistance and resilience to Alzheimer’s disease pathology are associated with reduced cortical pTau and absence of limbic-predominant age-related TDP-43 encephalopathy in a community-based cohort. *Acta Neuropathol Commun*. 2019;7(1):91. doi:10.1186/s40478-019-0743-1

21. Mahajan UV, Varma VR, Griswold ME, et al. Dysregulation of multiple metabolic networks related to brain transmethylation and polyamine pathways in Alzheimer disease: A targeted metabolomic and transcriptomic study. *PLOS Med*. 2020;17(1):e1003012. doi:10.1371/journal.pmed.1003012

22. Singh A, Allen D, Fracassi A, et al. Functional Integrity of Synapses in the Central Nervous System of Cognitively Intact Individuals with High Alzheimer’s Disease Neuropathology Is Associated with Absence of Synaptic Tau Oligomers. *J Alzheimer’s Dis*. 2020;78(4):1661-1678. doi:10.3233/JAD-200716

23. Zolochevska O, Taglialatela G. Selected microRNAs Increase Synaptic Resilience to the Damaging Binding of the Alzheimer’s Disease Amyloid Beta Oligomers. *Mol Neurobiol*. 2020;57(5):2232-2243. doi:10.1007/s12035-020-01868-8

24. Fracassi A, Marcatti M, Zolochevska O, et al. Oxidative Damage and Antioxidant Response in Frontal Cortex of Demented and Nondemented Individuals with Alzheimer’s Neuropathology. *J Neurosci*. 2021;41(3):538-554. doi:10.1523/JNEUROSCI.0295-20.2020

25. Walker JM, Kazempour Dehkordi S, Fracassi A, et al. Differential protein expression in the hippocampi of resilient individuals identified by digital spatial profiling. *Acta Neuropathol Commun*. 2022;10(1):23. doi:10.1186/s40478-022-01324-9

26. Taddei RN, Sanchez-Mico MV, Bonnar O, et al. Changes in glial cell phenotypes precede overt neurofibrillary tangle formation, correlate with markers of cortical cell damage, and predict cognitive status of individuals at Braak III-IV stages. *Acta Neuropathol Commun*. 2022;10(1):72. doi:10.1186/s40478-022-01370-3

27. Fracassi A, Marcatti M, Tumurbaatar B, Woltjer R, Moreno S, Taglialatela G. TREM2-induced activation of microglia contributes to synaptic integrity in cognitively intact aged individuals with Alzheimer’s neuropathology. *Brain Pathol Zurich Switz*. 2023;33(1):e13108. doi:10.1111/bpa.13108

28. Hurst C, Pugh DA, Abreha MH, et al. Integrated Proteomics to Understand the Role of Neuritin (NRN1) as a Mediator of Cognitive Resilience to Alzheimer’s Disease. *Mol Cell Proteomics MCP*. 2023;22(5):100542. doi:10.1016/j.mcpro.2023.100542

29. Taddei RN, Perbet R, Mate de Gerando A, et al. Tau Oligomer–Containing Synapse Elimination by Microglia and Astrocytes in Alzheimer Disease. *JAMA Neurol*. 2023;80(11):1209-1221. doi:10.1001/jamaneurol.2023.3530

30. Tumurbaatar B, Fracassi A, Scaduto P, et al. Preserved autophagy in cognitively intact non-demented individuals with Alzheimer’s neuropathology. *Alzheimers Dement J Alzheimers Assoc*. 2023;19(12):5355-5370. doi:10.1002/alz.13074

31. Guptarak J, Scaduto P, Tumurbaatar B, et al. Cognitive integrity in Non-Demented Individuals with Alzheimer’s Neuropathology is associated with preservation and remodeling of dendritic spines. *Alzheimers Dement*. 2024;20(7):4677-4691. doi:10.1002/alz.13900

32. Jury-Garfe N, Redding-Ochoa J, You Y, et al. Enhanced microglial dynamics and a paucity of tau seeding in the amyloid plaque microenvironment contribute to cognitive resilience in Alzheimer’s disease. *Acta Neuropathol (Berl)*. 2024;148(1):15. doi:10.1007/s00401-024-02775-1

33. Marcatti M, Tumurbaatar B, Zhang WR, et al. A brain‐derived tau oligomer polymorph is associated with cognitive resilience to Alzheimer’s disease. *Alzheimers Dement*. 2025;21(8):e70550. doi:10.1002/alz.70550

34. Jamison D, Kadamangudi S, Tumurbaatar B, et al. Comparative analysis of brain-derived tau oligomer interactomes in Alzheimer’s disease, non-demented with Alzheimer’s neuropathology, and primary age-related tauopathy: Implications for neurodegeneration and cognitive resilience. *J Alzheimers Dis*. 2025;106(4):1486-1508. doi:10.1177/13872877251352382
